# Epidemiological study of injuries in artistic swimming: a systematic review

**DOI:** 10.3389/fspor.2025.1509081

**Published:** 2025-02-25

**Authors:** Ane Begoñe Rincón, Alfonso Trinidad, Alejandro López-Valenciano

**Affiliations:** ^1^ Aqualab Research Group, Department of Education and Educational Innovation, Faculty of Law, Education and Humanities, European University of Madrid, Madrid, Spain; ^2^ Physical Exercise and Performance Research Group, Department of Education Science, School of Humanities and Communication Sciences, Universidad Cardenal Herrera-CEU, Castellon de la Plana, Spain

**Keywords:** prevention, sport, incidence, artistic, swimming

## Abstract

**Introduction:**

Artistic swimming is a highly technical sport that requires a large volume of training and forced positions that generate a high risk of injury.

**Objective:**

to compile scientific evidence on the incidence of injuries in artistic swimming. Literature study: PubMed, Web of Science, and SPORTDiscus databases were used to search for studies that analysed the epidemiology of injuries in artistic swimmers of any age and gender until June 2024. Methodology: the methodological quality of the studies was analysed with the Strengthening the Reporting Scale of Observational Studies in Epidemiology (STROBE) and Preferred Reporting Items for Systematic Reviews and Meta-Analyses guidelines (PRISMA) were followed. Synthesis: eleven studies met the inclusion criteria showing a clear trend of joint-ligament or muscle-tendon injuries in the shoulders, back, and knees.

**Conclusions:**

despite the publication of an injury surveillance document and a consensus on data collection and injury surveillance, there are methodological limitations that do not allow firm conclusions to be drawn. More epidemiological studies that follow data collection and injury surveillance guidelines are needed to establish differences by gender, age groups, and test.

## 1 Introduction

Artistic swimming is a sport whose practice and popularity has been growing in recent years, increasing the physical and psychological demands, and causing swimmers to acquire an increasingly athletic profile (1). Unlike other sports, it combines swimming, gymnastics, dance and ballet. Therefore, the requirements for strength, endurance, and flexibility, as well as the levels of execution technique, are greater to achieve performance (2). This implies great physical and mental exhaustion in collective and individual training, which can increase the risk of injuries. Injuries have a negative impact on swimmers, sometimes generating chronic pain or even the need for surgery, which causes a high number of absences from training and/or competitions (3). Additionally, in some cases, it can cause disability or withdrawal from the sport. Therefore, the development of effective preventive measures to reduce injuries is imperative to maintain the performance and health of the athlete in this discipline (2).

As in all aspects of sports medicine, knowledge of the epidemiology of injuries within a sports discipline (incidence, location, type, severity) allows establishing individualised guidelines and strategies, according to specific needs (4). Several articles have described that the most common injuries in artistic swimming occur due to overuse of the knee, shoulder, and lumbar spine, due to the nature of repetitive movements in training (2, 5). Likewise, since 2015, there has been a consensus on the recording of injuries in aquatic sports (6) that can help understand and control the epidemiology of injuries in artistic swimming. However, research on the prevalence of injuries in aquatic sports, such as swimming, has been quite precarious to date from a methodological point of view (7), which could also be reflected in other aquatic disciplines, such as artistic swimming, making it difficult to apply effective preventive programs.

For all these reasons, it is considered necessary to compile information to understand the epidemiology of the injuries and confirm whether the consensus established by Mountjoy et al. (8) in 2015 is being fulfilled. Along these lines, the main objective of the study was to compile scientific evidence on the incidence of injuries in artistic swimming, which does not exist to date to the authors’ knowledge and allows the creation of individualised and effective preventive programs.

## 2 Method

A systematic review of injuries in artistic swimming was conducted to compile scientific evidence on the incidence of injuries in artistic swimming (9). To carry out this study, the Preferred Reporting Items for Systematic Reviews and Meta-Analyses guidelines (PRISMA) (10) were followed.

### 2.1 Search strategy

Studies were identified by searching different data bases, including PubMed, Web of Science and SPORTDiscus, with the following search terms, combined through Boolean markers: (artistic swimming OR aquatic OR synchronised swimming) AND (injury OR incidence OR prevalence OR epidemiology); or in Spanish (artistic swimming OR aquatic OR synchronised swimming) AND (injury OR incidence OR prevalence OR epidemiology). The search was limited to June 30, 2024, and the languages included were English and Spanish. Subsequently, another complementary source, such as Google Scholar, was used, and manual searches were carried out in the selected studies to identify potentially eligible research not collected by the previous electronic searches.

Studies had to meet the following criteria: (1) had to report the incidence or prevalence of injuries in female artistic swimming swimmers; (2) published in a peer-reviewed journal; and (3) written in English or Spanish. Those investigations that (1) did not provide specific data on injuries in artistic swimmers, (2) were not published in peer-reviewed journals and (3) were written in languages other than Spanish and English were excluded. Likewise, (4) summaries, (5) literary reviews, (6) editorial comments, (7) letters to the editors and (8) studies with incomplete or inconsistent data were excluded. To guarantee the maximum possible objectivity, a code book was prepared to specify the characteristics of the studies (which can be obtained from the corresponding author).

The moderating variables of the studies were coded and grouped in to three categories: (1) general study descriptors (e.g., authors, country of sample, year of publication, and study design); (2) description of the study population (e.g., simple size, age, sex, level of swimmers); and (3) main epidemiological findings (e.g., injury and exposure data, distribution of injuries by anatomical location, type of injury, severity of injury, and mechanism of injury). For the storage and organisation of the selected studies, a data base was generated that can be obtained from the corresponding author.

On the other hand, two reviewers independently examined the title and abstract of each reference to locate potentially relevant studies. Once the screened documents were obtained, they reviewed them in detail to identify the articles that met the established criteria. A third external reviewer was consulted to resolve discrepancies between reviewers in the selected studies.

### 2.2 Quality evaluation

Two reviewers independently assessed the quality of included studies using an adapted version of the 'Strengthening the Reporting of Observational Studies in Epidemiology’ (STROBE) statement (11). The studies were scored according to 11 specific criteria that were derived from items 5, 6, 7, 8, 9, 12, 14, and 15 of the original checklist, and which have already been used in previous sports epidemiology studies (7, 11). The STROBE scale was considered an appropriate starting point for evaluating the quality of observational studies. This 11-item checklist provided guidance on the presentation of observational studies to facilitate the evaluation and interpretation of results. Likewise, observational studies were considered at low risk of bias if they were determined to be of high quality (score ≥7/11) or at high risk of bias if they were of low quality (≤6/11) (11).

Two researchers conducted the search and coding process for the articles to assess the inter-reviewer reliability of the coding process. For the qualitative variables, Cohen's kappa coefficients were applied. On average, the Kappa coefficient was 0.86 (range: 0.81–0.97), which can be considered very satisfactory. Inconsistencies between the two coders were resolved by consensus, and when the inconsistency was due to data base ambiguity, it was corrected. Any disagreements were resolved by mutual consent in consultation with a third review author.

## 3 Results

**Figure 1:**
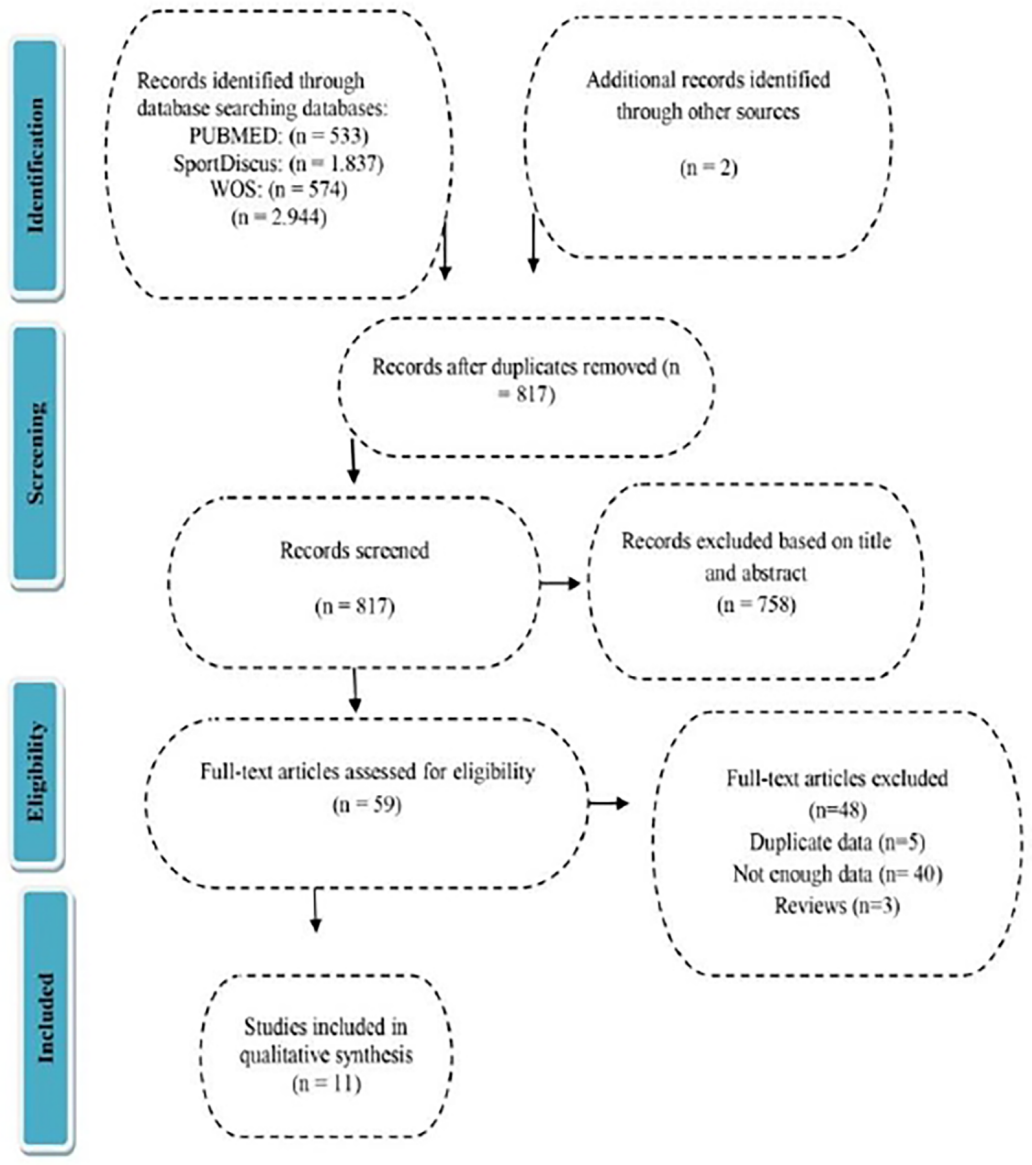
Flowchart of study selection in the systematic review.

After the search process, a total of 2,946 studies were found, of which 2,887 were eliminated due to non-compliance with the inclusion criteria, removal of duplicate articles, and review of the article title and abstract. The remaining 59 articles were selected for full review. Finally, 48 articles were excluded due to insufficient data or important methodological issues. The selection process can be found in Figure 1.

### 3.1 Descriptive characteristics of the studies

The main characteristics of the included studies are presented in Tables 1 and 2. The selected studies were carried out between 1991 and 2023. Two studies were carried out in Canada (15, 19), one study in Korea (14), one in France (20), and three collected information about athletes from all over the world as they participated in international competitions, such as world championships or the Olympic Games (3, 12, 13, 16, 17, 21, 22). Ten of the 11 studies included only women athletes, with only one study with mixed participation at the 2015 World Championships with 10 men (3). This study did not provide separate data on gender. Likewise, nine of the 11 studies used elite or high-performance athletes, and two used amateur-level athletes, they competed only in national and/or regional championships (19, 20).

**Table 1:** Characteristics of the studies included in the systematic review.

References	Level of participants	Athletes	Sex	Age (M ± SD or range)	Teams	Country	Year study
Engebretsen et al. (12)	Elite Olympic Games	104	Female	–	24	World	2012
Junge et al. (13)	Elite Olympic Games	104	Female	–	24	World	2008
Kim et al. (14)	Elite	51	Female	19,1 **±** 1,9	NA	Korea	2012–2019
Kirkley (15)	Elite junior and senior	85	Female	15–17	9	Canada	1988
Mountjoy et al. (16)	World Championship	281	Female	–	41	World	2009
Mountjoy et al. (6)	World Championship	306	Female	20,1 **±** 3,5	NA	World	2013
Prien et al. (3)	Elite Olympic Games	162	Mixed	20,9	21	World	2015
Soligard et al. (17)	Elite Olympic Games	104	Female	–	24	World	2016
Soligard et al. (18)	Elite World Championship	104	Female	–	24	World	2021
Steacy (19)	National level	1,151	Female	9–17	NA	Canada	2017–2018
Vignaud et al. (20)	National level	124	Female	12,9 **±** 1,6 (9–19)	1	France	2010–2020
	Future	32	Female	11,1 **±** 0,35 (<12)	1	France	2010–2020
	Youth	79	Female	13,1 **±** 1 (12–15)	1	France	2010–2020
	Junior	13	Female	15,8 **±** 0,83 (15–19)	1	France	2010–2020

NA, not available.

The hyphen that appears in some boxes means that this information is not provided by the authors.

**Table 2:** Injury definitions.

Reference	Injury definition
Engebretsen et al. (12)	An injury was defined as new or recurring musculoskeletal complaints or concussions (injuries) receiving medical attention, regardless of the consequences with respect to absence from competition or training.
Junge et al. (13)	An injury was defined as any musculoskeletal complaint (traumatic and overuse) newly incurred due to competition and/or training that received medical attention regardless of the consequences with respect to absence from competition or training.
Kim et al. (14)	An injury was defined as any acute or chronic musculoskeletal signs or symptoms occurring due to aquatic activities during training sessions.
Kirkley (15)	Any disability requiring alteration or cessation of training.
Mountjoy et al. (16)	An injury was defined as any musculoskeletal complaint and/or concussion newly incurred due to competition and/or training that received medical attention regardless of the consequences with respect to absence from competition and/or training.
Mountjoy et al. (6)	An injury was defined as any musculoskeletal complaint and/or concussion newly incurred due to competition and/or training that received medical attention regardless of the consequences with respect to absence from competition and/or training.
Prien et al. (3)	Health complaints were defined as any physical or mental health disorder, regardless of the consequences for participation in training or competition.
Soligard et al. (17)	An injury was defined as new or recurring musculoskeletal complaints or concussions (injuries) receiving medical attention, regardless of the consequences with respect to absence from competition or training.
Soligard et al. (18)	An injury was defined as new or recurring musculoskeletal complaints or concussions (injuries) receiving medical attention, regardless of the consequences with respect to absence from competition or training.
Steacy (19)	Concussion: It is the result of pathological metabolic changes in the brain, induced by biomechanical forces attendant to a direct or indirect blow to the head.
Vignaud et al. (20)	The injuries were defined by a physical complaint reported by an athlete about an injury occurring during competition or training and requiring medical attention.

### 3.2 Epidemiology of injuries

Three of the 11 selected studies included incidence values in their research (14, 20, 22). Kim et al. (14) in 2020 and Vignaud et al. (20) in 2023 showed an incidence of 2.0 and 1.05 injuries per-1,000 h of training, while Soligard et al. (2023) revealed that 5.8 Olympic artistic swimmers suffered injuries per-100 athletes. Injuries were recorded using daily reports completed by the medical team or retrospective questionnaires. Apart from these three studies (14, 19, 20), all studies had a short duration of injury recording (four weeks or less). In total, 432 injuries were identified in the investigations selected for this systematic review of artistic swimming. All data are detailed in Table 3.

**Table 3:** Epidemiology of injuries in artistic swimming.

Reference	Injury surveillance	Follow-up weeks	Total exposure (hours)	Training exposure (hours)	Injuries	Training injuries	Competition injuries	Incidence	Training incidence	Competition incidence
Engebretsen et al. (12)	Report daily physicians	1 week	–	–	14	–	–	–	–	–
Junge et al. (13)	Report daily physicians	1 week	–	–	2	2	0	–	–	–
Kim et al. (14)	Injury report daily	8 years	–	81,896	164	164	0	–	2.0	–
Kirkley (15)	Questionnaire	NA	–	–	53	–	–	–	–	–
Mountjoy et al. (16)	Report daily physicians	1 week	–	–	28	–	–	–	–	–
Mountjoy et al. (6)	Report daily physicians	1 week	–	–	5	1	4	–	–	–
Prien et al. (3)	Questionnaire	4 weeks	–	–	36	–	–	–	–	–
Soligard et al. (17)	Report daily physicians	1 week	–	–	6	4	2	–	–	–
Soligard et al. (18)	Report daily physicians	1 week	–	–	6	5	1	5.8	–	–
Steacy (19)	Digital injury report	1 year	–	–	–	21	1	–	–	–
Vignaud et al. (20)	Retrospective questionnaire with physicians	11 years	–	112,503	–	118	–	–	1.05	–

The hyphen that appears in some boxes means that this information is not provided by the authors.

### 3.3 Location of the injury

Eight of the 11 selected articles provided information on the location of the injuries (12, 14–17, 20–22). Most studies indicated that the most prevalent injuries were in the shoulder and knee (14, 15, 17, 20, 22), followed by the lumbosacral area. Three studies showed other locations, such as the foot/toe (12), thigh (16), and hip/groin (21). In these three studies, shoulder injuries were also among the most prevalent. More detailed information can be obtained in Tables 4, 5.

**Table 4:** Location of injuries of the studies included in the review (trunk upwards).

Reference	Head and neck	Upper limbs	Shoulder	Arm/elbow	Wrist/hand	Trunk	Chest	Thoracic spine	Lumbosacral	Abdomen
Engebretsen et al. (12)	2	2	1	–	1	3	–	–	–	–
Kim et al. (14)	15	62	30	18	14	25	1	5	19	0
Kirkley (15)	–	25	21	4	–	4	–	–	–	–
Mountjoy et al. (16)	3	6	4	1	1	2	–	–	–	–
Mountjoy et al. (6)	–	2	1	1	–	–	–	–	–	–
Soligard et al. (17)	1	1	1	–	–	2	–	–	–	–
Soligard et al. (18)	1	0	–	–	–	2	–	–	–	–
Vignaud et al. (20)	–	43	23	10	10	16	–	–	16	–
	–	7	1	3	3	2	–	–	2	–
	–	26	17	6	3	14	–	–	14	–
	–	10	5	1	4	0	–	–	0	–

The hyphen that appears in some boxes means that this information is not provided by the authors.

**Table 5:** Location of injuries of the studies included in the review (lower limbs).

Reference	Lower limbs	Hip/groin	Thigh	Knee	Lower leg/achilles tendon	Ankle	Foot/toe	Undefinied/other
Engebretsen et al. (12)	7	–	1	–	–	–	6	–
Kim et al. (14)	62	3	13	12	5	21	8	–
Kirkley (15)	17	–	–	17	–	–	–	5
Mountjoy et al. (16)	13	2	5	2	–	4	–	4
Mountjoy et al. (6)	3	3	–	–	–	–	–	–
Soligard et al. (17)	1	–	–	1	–	–	1	–
Soligard et al. (18)	3	–	–	2	1	–	–	–
Vignaud et al. (20)	59	9	4	29	12	4	1	–
	18	2	1	9	5	1	0	–
	38	7	3	18	6	3	1	–
	3	0	0	2	1	0	0	–

The hyphen that appears in some boxes means that this information is not provided by the authors.

### 3.4 Type of injury

Six studies analysed the type of injuries suffered by artistic swimmers (12, 14, 16, 17, 19, 22). Joint (non-bone) and ligament injuries were the most prevalent in five of the seven studies. Only Mountjoy et al. (16) in 2010 showed more muscle-tendon injuries, followed by joint (non-bone) and ligament injuries. On the other hand, Steacy (19) in 2019 analysed only concussions suffered over the course of a year. However, all these statements should be analysed with caution due to the different classifications used in each study. Table 6 presents the type of the injuries per study.

**Table 6:** Type of injury of the studies included in the systematic review.

Reference	Concussions	Fractures and bone stress	Joint (non-bone) and ligament	Mucle and tendon	Contusions	Laceration and skin lesion	Undefinied/other
Engebretsen et al. (12)	–	2	4	2	1	1	4
Kim et al. (14)	–	–	93	71	–	–	–
Mountjoy et al. (16)	–	1	6	9	3	1	8
Soligard et al. (17)	–	–	4	1	1	–	–
Soligard et al. (18)	–	–	2	1	2	1	–
Steacy (19)	22	–	–	–	–	–	–

The hyphen that appears in some boxes means that this information is not provided by the authors.

### 3.5 Injury mechanism

Three studies provided information on the mechanism of injury (12, 17, 22). Two of the three studies showed that contact/traumatic injuries were slightly more prevalent than overuse injuries (12, 22). However, we must also consider overuse injuries, since the numerical difference recorded is not significant. One article showed the same number of injuries in both categories (17).

### 3.6 Injury severity

Only one study rigorously analysed injury severity (17), showing four minimal injuries (1–3 days) and two moderate injuries (8–28 days).

### 3.7 Quality of the selected studies

Regarding the quality of the studies, the average score obtained with the STROBE scale was 7.9 (minimum: 1, maximum: 10), showing medium-high quality, and there may be low bias in most of the studies included in the review. It should be noted that only five of the 11 articles collected included the FINA consensus regarding their injury registry (3, 14, 17, 21, 22). The data is presented in Table 7.

**Table 7:** Analysis of the selected studies methodological quality.

References	1	2	3	4	5	6	7	8	9	10	11	Total
Engebretsen et al. (12)	+	+	+	−	+	+	−	+	+	+	+	9
Junge et al. (13)	+	+	+	−	+	+	+	+	+	+	+	10
Kim et al. (14)	+	+	+	−	+	+	+	+	+	−	+	9
Kirkley (15)	+	−	−	−	−	−	−	−	+	−	+	3
Mountjoy et al. (16)	+	+	+	−	+	+	+	−	+	+	+	9
Mountjoy et al. (6)	+	+	+	−	+	+	−	+	+	+	+	9
Prien et al. (3)	+	+	+	−	+	+	−	+	+	−	+	8
Soligard et al. (17)	−	−	+	−	+	+	+	+	+	+	+	8
Soligard et al. (18)	−	−	+	−	+	+	+	+	+	+	+	8
Steacy (19)	+	+	+	−	+	+	+	+	−	+	+	9
Vignaud et al. (20)	+	+	−	−	−	+	−	−	+	−	+	5

The hyphen that appears in some boxes means that this information is not provided by the authors.

The numbers of the columns corresponded to the following items of the STROBE scale.

*Materials and methods.*

1. Describes the setting or participating locations.

2. Describes relevant dates (period of recruitment, exposure, follow-up, data collection).

3. Provides statement concerning institutional review board approval and consent.

4. Gives the inclusion and exclusion criteria.

5. Describes injury history.

6. Describes methods of follow-up.

*Data sources/measurement.*

7. Provides a definition of injury.

8. Verifies injury by an independent medical professional.

9. Classifies injury (severity, location and type of injury).

10. Indicates the number of participants with missing data and explain how this was addressed.

11. Measures and presents exposure data.

## 4 Discussion

The objective of the present study was to collect scientific evidence on the incidence of injuries in artistic swimming, so that sports science professionals can propose clear and concise objectives in their preventive programs. The main finding of this review was that, to date, methodological recommendations for data collection and injury surveillance in aquatic sports have not been followed (21), showing great heterogeneity between studies or compiling only some points of injury epidemiology (incidence, location, type, mechanism, and severity of injuries). Only five studies included and used these methodologies in their research (3, 14, 17, 21, 22) and they also did not provide information in all categories of injury epidemiology.

Additionally, several injury definitions and different classifications were used. Consequently, this lack of methodological guidelines made it difficult to compare all these results that would allow in the future to establish homogeneous and effective injury prevention guidelines. Therefore, it is relevant to change the trend of data collection on the epidemiology of injuries in artistic swimming and follow the established guidelines. Following this line, future recommendations should emphasize the need for a standardized injury surveillance protocol, especially considering the low number of studies that adhered to the FINA consensus.

At an epidemiological level, injuries to the shoulder, knee, and trunk (more specifically the lumbar spine) are the most common among artistic swimmers and could be considered the main locations of injury. Also worth noting is the lack of exposure hours recorded in total; only two studies had exposure data during training (14, 20). Regarding the type of injury, like most sports, muscle-tendon and joint-ligament injuries prevail. Seeing all this data, it would be very interesting to create research around the creation of preventive programs based on locations and types of injuries.

It is important to highlight that the use of different training techniques and different environments could mitigate the appearance of injuries (22, 23). This, taken to artistic swimming, could be interesting, since if strength or flexibility exercises are performed in different environments in the most affected areas, injuries could reduce its appearance.

### 4.1 Injury incidence

Soligard et al. (18) revealed that 5.8 Olympic artistic swimmers suffered injuries per-100 athletes. Added to that data, Kim et al. (14) and Vignaud et al. (20) found an incidence of 2.0 and 1.05 in artistic swimming training, respectively. These data show a similar incidence like other aquatic sports. Chase et al. (7) showed an incidence of 3.04 injuries/1,000 h, 5.55 injuries/1,000 h or even 2.6 injuries/1,000 h in swimming. Likewise, in diving there is an incidence of 2.61 (14). This may be mainly due to the characteristics of the environment in which these sports are developed, with little impact, without interference from other rivals and without any implements, which greatly reduces the probability that athletes may suffer some type of injury (2).

### 4.2 Injury location

Artistic swimmers train their cardiorespiratory capacity by swimming mainly in freestyle, which adds to the repetition of the skills inherent to the discipline (arm actions in routines, rowing, and exercises on dry land) in training, and the large volume of hours of training (24), could explain shoulder overload. During these trainings, repetitive cyclic movement of the glenohumeral joint occurs (25). These repetitive and continuous movements generate great fatigue and friction between the different structures of the joint, which result in microtrauma to the rotator cuff, particularly the supraspinatus and sometimes the long head of the biceps (2), thus causing inflammation and pain (26). Along the same lines, fatigue caused by pain generates a biomechanical alteration in shoulder movement that can trigger an injury (18, 27). Therefore, the repetitive practice of these actions for several hours daily could increase the risk of shoulder injury in artistic swimmers (5).

Regarding knee injuries, a conditioning factor is the mixer kick that the swimmer uses to stay on the water's surface, while the arms move in the air (5). As it is a repetitive and widely used movement, it could be stated that the cause of the injury is mainly due to it excessive use (28). Furthermore, the frequency and technical intensity of the kick (29), together with a decompensation of the knee angle or a mechanical imbalance of the hip joint during it (29), could affect the movement pattern of this joint and cause medial joint pain (due to knee valgus) or anterior joint pain (due to abnormal tracking of the patella in the trochlear not chop the femur), which would increase the risk of injury (2). Therefore, the importance of teaching the technical execution of the beater kick and the breaststroke kick among swimmers is highlighted, in addition to carrying out adequate preventive work to avoid possible propulsive irregularities at a biomechanical level among athletes to prevent possible injuries.

Likewise, trunk and spine injuries could lie in errors made during training, such as excessive repetitions, explosive speeds, arching with an excessive rotational component, inadequate neuromuscular training, poor stability, core strength and posture, as well as poor flexibility training (2). These errors have generated an increase in the prevalence of low back pain syndrome due to repetitive and rapid arching (5). Some movements performed during warm-ups have also required that the lumbar spine be maintained in hyperextension, as this achieves a streamlined body position; this can place excessive load in the lower back (27). Consequently, teaching, correcting and maintaining good swimming technique, modifying the frequency, strength, speed, and degree of hyperextension in the acute phase (5), as well as strengthening the lumbopelvic complex area (30), could help reduce the risk of injuries, especially in the trunk and lumbar spine of artistic swimmers (2, 31). Therefore, physiotherapists and physical exercise professionals should integrate this methodology into their training programs to improve the sports performance of swimmers and there by prevent possible injuries (32).

### 4.3 Injury type

Muscle-tendon and joint (non-bone) and ligament injuries are closely associated with the location and mechanism of the injuries mentioned above, since they are a consequence of rapid and repetitive movements in the areas of the shoulder, knee, and trunk (lumbosacral) (27, 28). For example, the repetitive motion of the synchronised kick causes great stress on the ligaments and tendons of the knee joint (27, 29). Likewise, the literature has found spinal disorders, such as scoliosis, hyperlordosis, hyperkyphosis, and low back pain syndrome (2). The need to maintain the most aerodynamic and efficient position possible through hyperextension, together with the constant rotation and arching of the trunk, generates a great overload in the lumbar area that could give rise to different tendon, ligament, or muscular pathologies (5, 27).

On the other hand, the high number of bruises and concussions observed in the study by Steacy (19) could be due to the performance of some stunts (33). Therefore, it could be stated that special attention to the correct technical execution of specific elements of artistic swimming (such as the beater kick, positions with lumbar hyperextension, and acrobatics), greater strengthening of the muscles involved, together with a program to prevent ligament, muscular, and tendon injuries, it could reduce the risk of injuries in artistic swimmers.

However, this entire statement must be analysed with caution due to the different classifications used in each study and the lack of existing information to draw meaningful conclusions. Therefore, future lines of work should focus on improving the methodology following the consensus on the recording of injuries in aquatic sports (21). Furthermore, it is strongly recommended to exhaustively record hours of exposure to obtain incidence data for comparison with other similar modalities, and to include all injury patterns in the studies (location, type, mechanism and severity of injury). These common data presentation practices would allow the development of specific preventive programs that can reduce the risk of the most common injuries in artistic swimming.

## 5 Limitations

As there are not many scientific publications in the literature, few studies were compiled that provided relevant information on the severity and mechanism of injury in different age groups, thus limiting validity and reliability when creating preventive, effective, and individualised programs. Only studies in English and Spanish were searched, so perhaps, other documents from Japan and Russia, with a strong artistic swimming, could not be included. Finally, it should be noted that there are no studies that compare and explain different risk patterns by gender, age, or skill level, despite evidence of anatomical, biomechanical, and physiological differences. Therefore, it is a notable limitation that in this study we have not been able to perform the classification based on these variables.

## 6 Conclusions

At an epidemiological level, it does seem clear that the main injuries in artistic swimming are joint and ligament, and muscle/tendinous, especially in the shoulder, knee, and trunk (lumbosacral). Therefore, to avoid injuries in artistic swimming swimmers, it is advisable to analyse the most frequent injuries, strengthen the affected areas and establish a prevention plan following the FINA consensus. However, there is not enough information due to methodological limitations and the results should be taken with caution.

## Data Availability

The original contributions presented in the study are included in the article/Supplementary Material, further inquiries can be directed to the corresponding author.
